# Case Report: Perioperative Kounis Syndrome in an Adolescent With Congenital Glaucoma

**DOI:** 10.3389/fcvm.2021.676188

**Published:** 2021-09-10

**Authors:** Guglielmo Capponi, Mattia Giovannini, Ioanna Koniari, Francesca Mori, Chiara Rubino, Gaia Spaziani, Giovanni Battista Calabri, Silvia Favilli, Elio Novembre, Giuseppe Indolfi, Luciano De Simone, Sandra Trapani

**Affiliations:** ^1^Cardiology Unit, Department of Pediatrics, Meyer Children's University Hospital, Florence, Italy; ^2^Allergy Unit, Department of Pediatrics, Meyer Children's University Hospital, Florence, Italy; ^3^Electrophysiology and Device Department, University Hospital of South Manchester NHS Foundation Trust, Manchester, United Kingdom; ^4^Department of Pediatrics, Meyer Children's Hospital, Florence, Italy; ^5^Department of NEUROFARBA, Meyer Children's Hospital, University of Florence, Florence, Italy; ^6^Department of Health Sciences, Meyer Children's Hospital, University of Florence, Florence, Italy

**Keywords:** Kounis syndrome, perioperative, midazolam, sevoflurane, coronary artery, pediatrics

## Abstract

A 12-year-old male patient suffering from congenital glaucoma developed bradycardia, left ventricular failure, and hypotension after induction of anesthesia. Electrocardiography and echocardiography revealed a complete normalization of ECG and a complete spontaneous recovery in the cardiac function 72 hours from the beginning of the clinical manifestations, while cardiac Magnetic Resonance Imaging was performed, and coronary Computed Tomography scan revealed a myocardial bridge of a tract of the left anterior descendent coronary artery. Diagnosis of Kounis syndrome (KS) was made, a relatively novel, under-recognized clinical condition, defined as the manifestation of an acute coronary syndrome accompanied by mast cell activation and platelet aggregation involving interrelated and interacting inflammatory cells in the setting of allergic, hypersensitivity, anaphylactic or anaphylactoid insults. We described one of the first pediatric cases of KS related to anesthetic medications. In children, this syndrome has been only described in isolated case reports or small case series. Thus, it appears critical to report new cases of KS in children to increase the awareness of this disease in pediatric healthcare workers so as to enhance its early recognition and optimal therapeutic strategy. Furthermore, it appears of paramount importance the implementation of universal guidelines accepted by allergology and cardiology societies, in order to standardize the management of pediatric and adult patients with KS. Finally, a close collaboration between pediatric allergists and cardiologists seems fundamental for an optimal multidisciplinary patient care.

## Introduction

Kounis syndrome (KS) is defined as the manifestation of an acute coronary syndrome accompanied by mast cell activation and platelet aggregation involving interrelated and interacting inflammatory cells in the setting of allergic, hypersensitivity, anaphylactic or anaphylactoid insults (1, 2). However, the exact physiopathology of this syndrome has not been fully elucidated.

Although this condition could potentially occur in every age, only a minority of cases (9.1%) are described in subjects younger than 20 years of age compared to older ages (68.0% at 40–70 years old) (3). The main reason could be attributed to an underestimation of this relatively novel syndrome in the pediatric age (4). Several triggers have been associated with KS, including drug administration, food consumption, and insect bites. Antibiotics (27.4%) and insect bites (23.4%) are demonstrated as the most common triggers eliciting this syndrome (1, 3). KS caused by anesthetic medications has already been reported as a cause of acute coronary syndrome (5–8). However, to the best of our knowledge, we describe one of the first pediatric cases of KS associated to anesthetic drugs (4, 8–14).

## Case Description

A 12-year-old male suffering from congenital glaucoma was admitted to Meyer Children's University Hospital for the surgical revision of his ocular Baerveldt implant, a device that maintains a patent connection between the anterior chamber of the eye and an equatorial bleb capsule and improves the drainage of the aqueous out of the eye (15). This patient suffered from a pathologic mutation on CYP1B1, encoding a dioxin-inducible enzyme belonging to the cytochrome P450 superfamily, that is involved in the proper development and balance of trabecular meshwork (16). Because of this chronic ocular disease, the patient had previously undergone other surgical procedures with no reported anesthesiologic complications. A previous surgery was performed 3 months before and the anesthesia was administered with inhaled sevoflurane and intravenous fentanyl, accompanied with intravenous ondansetron as antiemetic drug, without any clinical reaction. His past medical history was negative for any cardiac issue or atopic condition, and his family medical history was unremarkable.

About 30 minutes after the induction phase of the anesthesia with intravenous midazolam bolus, followed by continuous inhaled sevoflurane, the patient experienced an episode of bradycardia associated with hypotension, apparently without reporting any clinical signs and symptoms (dyspnoea, chest pain, pulmonary or peripheral oedema). Specifically, his heart rate decreased from 90 to 40 bpm; however, this clinical manifestation seemed to respond to the administration of a bolus of atropine that further induced a transient episode of sinus tachycardia. At the same time, his blood pressure dropped to 80/45 mmHg. In the acute phase, the physical examination revealed a mild apical systolic murmur (2/6 on the Levine grading scale), without any other clinical manifestations of heart failure.

**Figure 1:**
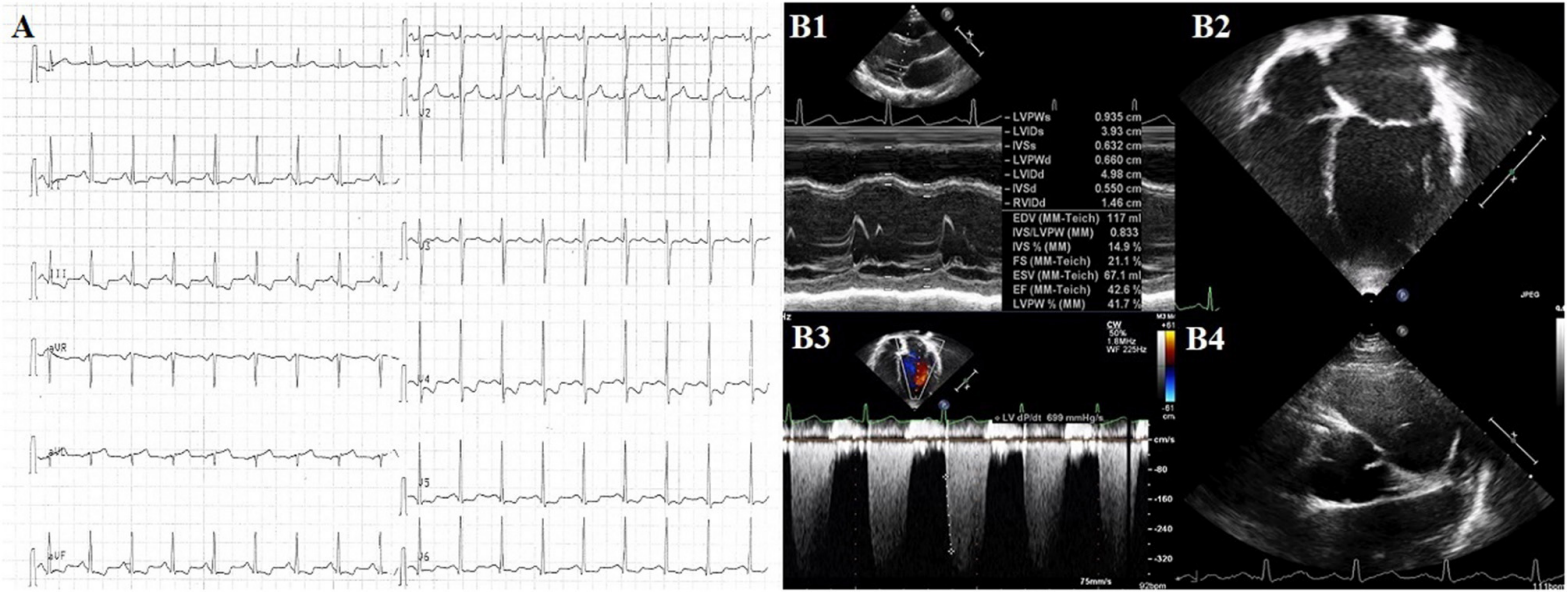
First electrocardiogram and echocardiogram after the onset of the clinical manifestations **(A)**. The electrocardiogram shows sinus rhythm with 100 rpm, PR 0.14 s, QRS 0.08 s, QT/QTc 320/422 ms and diffuse alterations in ventricular repolarization with negative T waves in precordial leads (V4–V6), D2, D3 and aVF **(B1–B4)**. The echocardiogram shows a diffusely hypokinetic left ventricle with an ejection fraction of 34%. Left ventricular inner dimension diastole 50 mm (Z score +2.3), left ventricular inner dimension systole 39 mm (Z score +4.25), left posterior ventricular wall diastole 6.6 mm (Z score −0.23), interventricular septum diastole 5.5 mm (Z score −1.22), right ventricular inner dimension diastole 14 mm (Z score +0.20) [Z score is derived from Kampman et al. (17)] **(B1,B2)**. Moderate mitral valve regurgitation (left ventricular delta Pressure/delta Time 699 mmHg/s) **(B3)**. Left and right coronary artery originating from left and right Valsalva sinus, respectively **(B4)**.

An electrocardiogram (ECG) showed sinus rhythm with ventricular repolarization abnormalities in the inferior lateral wall (Figure 1). Moreover, the echocardiographic study demonstrated global left ventricular (LV) hypokinesia and dilatation with preserved right ventricular function (Figure 1). Laboratory work-up documented an increase of creatine kinase-myocardial band (CK-MB) and troponin. Furthermore, N-terminal-prohormone B-type natriuretic peptide (NT-proBNP) was increased as well, consistent with LV dysfunction. Further blood investigations did not show any inflammatory markers increase, eosinophilia, electrolytic imbalance, and liver, or kidney dysfunction (Table 1).

**Table 1:** Laboratory tests trend from the admission to the discharge of the patient.

	**Admission**	**2 days**	**4 days**	**Discharge**	**Normal**
		**after admission**	**after admission**		**values**
WBC (x 10^3^/μL)			6.36		4.10–12.00
RBC (x 10^6^/μL)			4.66		4.30–5.80
Hb (g/dL)			13.7		12.60–17.00
PLT (x 10^3^/μL)			230		190–460
N (%)			54.4		35.0–65.0
L (%)			32.7		15.0–55.0
M (%)			7.9		2.0–15.0
E (%)			2.6		0.0–6.0
B (%)			0.5		0.0–3.0
CRP (mg/dL)	<0.290				0.0–0.5
Na (mEq/L)			139		133–145
K (mEq/L)			3.8		3.3–5.2
Cl (mEq/L)			104		95–110
AST (IU/L)			26		8–60
ALT (IU/L)			31		7–55
Creatinine (mg/dL)	0.56				0.40–0.80
CK (IU/L)	86	81			20–200
CK-MB (ng/mL)	7.5	1.3			0.5–3.6
Troponin (μg/L)	2.03	0.21	0.14		0.00–0.09
NT-proBNP(pg/mL)	830	1,300	1,835	601	5–242

*ALT, alanine transaminase; AST, aspartate transaminase; B, basophils; CK, creatine kinase; CK-MB, creatine kinase-myocardial band; Cl, chloride; CRP, C-reactive protein; E, eosinophils; Hb, hemoglobin; K, potassium; L, lymphocytes; M, monocytes; N, neutrophils; Na, sodium; NT-proBNP, N-terminal-prohormone B-type natriuretic peptide; PLT, platelets; RBC, red blood cells; WBC, white blood cells*.

The patient was hemodynamically stable, but a close follow-up was planned, including a daily electrocardiogram and echocardiography for prompt evaluation of any additional ECG-ischemic changes or cardiomyopathy deterioration.

**Figure 2:**
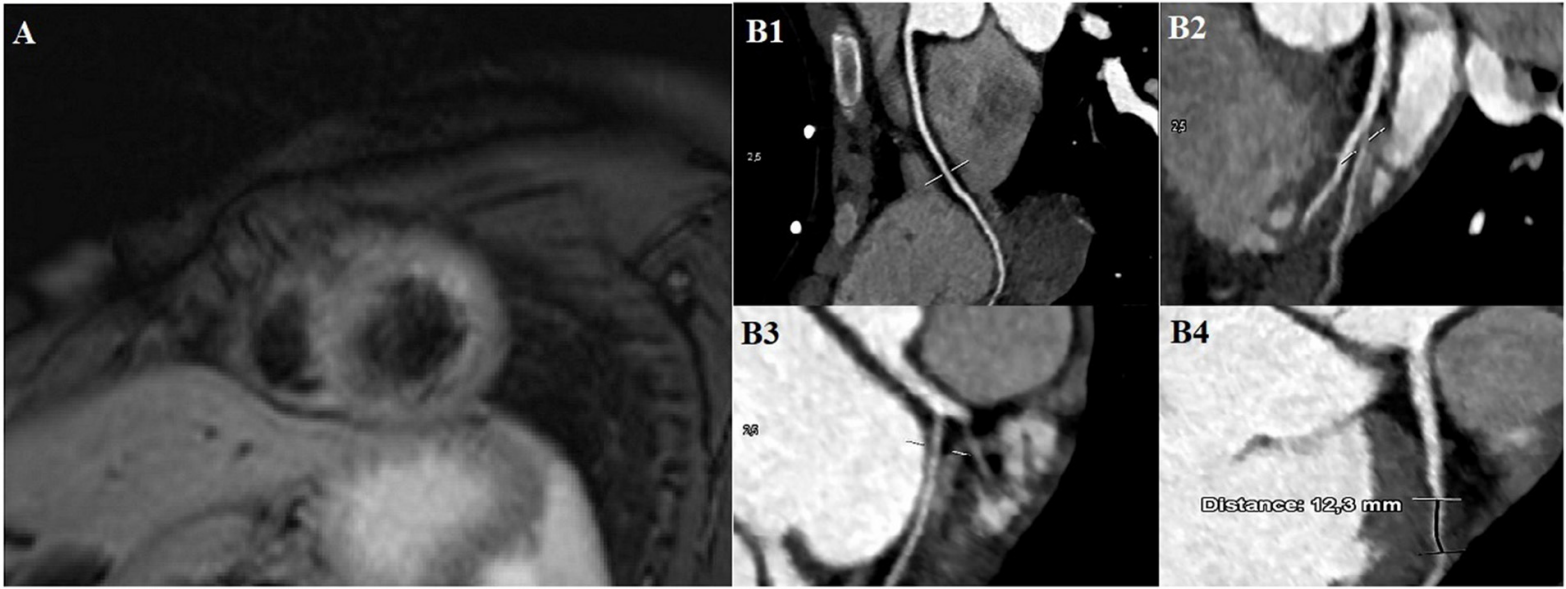
**(A)** Cardiac contrast-enhancement magnetic resonance imaging demonstrates an increase of T2 signal on anterior left ventricular wall. **(B1–B4)** Coronary computed tomography reveals a right dominant coronary artery **(B1)**, left common trunk coronary artery **(B2)**, left circumflex coronary artery **(B3)** and left anterior descending (LAD) coronary artery **(B4)** without any dissection, lumen obstruction or anatomical variants. A tract of LAD coronary artery runs through the width of the myocardial wall for 12.3 mm and with a parietal thickness of 1.4 mm **(B4)**.

**Figure 3:**
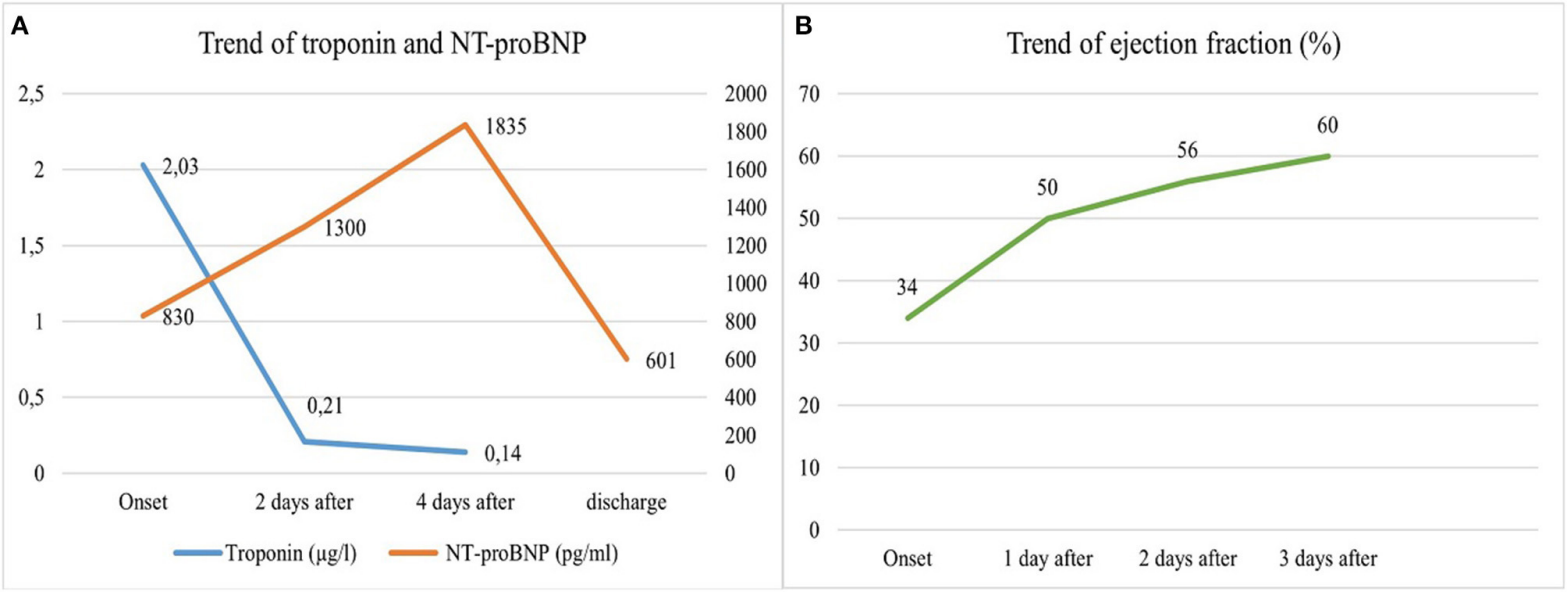
Trend of troponin, NT-proBNP **(A)** and ejection fraction **(B)** from the admission to the discharge of the patient. NT-proBNP, N-terminal-prohormone B-type natriuretic peptide.

Two days after admission, in order to exclude other causes of cardiomyopathy, such as myocarditis, a cardiac contrast-enhancement magnetic resonance imaging (CE-MRI) demonstrated a significant improvement of LV function without any regional wall abnormality. Oedema was noticed on the anterior ventricular wall (Figure 2), possibly consistent with reversible myocardial injury potentially related to myocardial ischemia, while no signs of fibrosis were demonstrated after gadolinium enhancement. A daily cardiac evaluation revealed a complete normalization of ECG and a complete recovery in the cardiac function 72 hours from the beginning of the clinical manifestations, along with a marked reduction in CK-MB, troponin and NT-proBNP, at discharge, 6 days after admission (Figure 3), while the patient was in good clinical condition, in absence of drug therapy.

A coronary computed tomography (CT), 10 days after the onset of the clinical manifestations, excluded any lumen obstruction, dissection or other anatomical variants of the aorta and coronary arteries. However, it was clearly observed myocardial bridge of a tract of the left anterior descending (LAD) coronary artery (Figure 2). As a precaution, it was chosen not to perform a provocative stress test during the acute phase of the disease, but in order to exclude further stress-induced ischemia an exercise test was performed 21 days after the onset of the clinical manifestations. It showed a linear increase in heart rate and blood pressure, without ischemic signs, symptoms or ST-T ECG alterations.

The patient underwent skin prick testing with midazolam (5 mg/ml) and sevoflurane (100%) with negative results. According to current standards, histamine 10 mg/ml (Lofarma, Milan, Italy) and normal saline were used as positive and negative control substances, respectively.

## Discussion

KS is a rare and poorly characterized entity in children (2, 4, 8–14). In adults, this condition could be associated with other factors, such as previous allergy, hypertension, smoking, diabetes and hyperlipidemia, whereas these features may be less important in children and adolescents (3, 18, 19). The clinical suspicion is based on clinical signs and symptoms (including acute chest pain, chest discomfort, dyspnoea, bradycardia, hypotension, pallor, palpitations or clinical manifestations of a reaction) associated with a potential trigger. KS diagnosis should be highly suspected when these clinical findings are accompanied by ECG signs (including atrial fibrillation, sinus bradycardia, sinus tachycardia, ST-segment elevation or depression, T-wave flattening or inversion), an increase of myocardial injury biomarkers or possible evidence of coronary spasm/thrombosis on coronary angiography (1).

The differential diagnosis of KS includes other conditions related to myocardial injury, including spontaneous coronary artery dissection, fibromuscular dysplasia, myocardial bridging, myocarditis and takotsubo syndrome (20, 21).

According to diagnostic criteria of myocarditis in our patient the diagnosis of infectious myocarditis could be excluded, due to the absence of fever or previous infectious clinical signs and symptoms, values of the blood tests, and findings of the MRI (22).

Sometimes, it can be difficult to distinguish takotsubo syndrome from KS and, as already reported, in some cases they can even coexist (23, 24). Interestingly, both these conditions share some common pathologic mechanisms: vascular hyperreactivity of coronary arteries and local/systemic cellular or humoral inflammatory response in inducing myocardial damage (25, 26).

In our patient, there was a close relationship between the administration of anesthetic medications and the onset of the clinical manifestations [probable association according to Naranjo scale (27)]. Furthermore, there was an increase of troponin and CK-MB, together with ECG abnormalities (diffuse repolarization abnormalities in inferior and lateral leads) and echocardiographic features (diffuse hypokinesia of LV wall). The latter characteristics strongly supported the diagnosis of KS.

Okur et al. defined the cardiac MRI criteria for KS type 1 diagnosis: early phase subendocardial contrast defect, T2 weight images showing high-intensity signal consistent with oedema of all lesion areas, lack of the late gadolinium enhancement, and no transmural involvement (28). This study reported different percentages of left-wall myocardial involvement with a prevalence in interventricular septum (53.8%), followed by LV lateral wall (30.7%). It is interesting to note that MRI in our patient highlighted an augmented T2 signal in the anterior LV wall without late gadolinium enhancement, probably supporting vasospasm in the left anterior descending coronary artery, concordant with the ECG data.

Coronary angiography is the gold standard to demonstrate the anatomy of coronary arteries and to exclude other causes of lumen obstruction. The coronary-CT of our patient demonstrated myocardial bridging of a tract of LAD coronary artery. Drug reactions and the narrowing of the coronary lumen due to myocardial bridging have already been reported as participating co-factors leading to myocardial ischemia (29). Indeed, it seems unlikely a direct role of a myocardial bridging as the unique cause of the transient myocardial damage, even if it could be accounted as a potential favoring condition.

In a recent retrospective analysis, allergic reactions due to sedative e/o analgesic medications during pediatric pre-procedural sedation were described with an incidence of about 1:4,219, with midazolam being the third most frequently reported drug (1:2,035) (30). Midazolam can also be associated with severe adverse events, for example, anaphylactic and anaphylactoid reactions (31–34). Moreover, an acute coronary syndrome has already been described in the literature as a side effect of this anesthetic drug in a previous case report of KS in an adult patient during the preoperative phase of a transurethral prostatectomy (5). Furthermore, inhaled anesthetic medications, such as isoflurane, have already been reported as a possible precipitating factor of acute coronary vasospasm in a 59-year-old man and in a 2-year-old boy (6, 8). Sevoflurane was used in our case and, of note, our patient had received anesthesia comprising sevoflurane without clinical reactions 3 months before. However, he was suggested to strictly avoid drugs potentially associated to the event in the future.

As occurred in our case, diagnosis of KS can be difficult when the patient is not able to report signs and symptoms. In such conditions the suspicion and the diagnosis establishment should be based mostly on the close association between a trigger, for example a drug, and the occurrence of ECG signs, the increase of myocardial injury biomarkers or possible evidence of coronary spasm/thrombosis on coronary angiography (1).

In anaphylaxis, cutaneous manifestations can lack in a minority of cases. In a cohort of adult and pediatric patients with this disease, skin was affected in 84% of them, followed by clinical manifestations involving cardiovascular (72%) or respiratory (68%) systems (35, 36). In severe anaphylaxis the phenomenon has been hypothesized due to the state of shock that prevents or delays the released anaphylactic mediators from reaching and acting on the skin, thus inducing redness, rash and/or itching (37, 38). The absence of skin lesions can also occur in KS, in which rash has been described in 26.8% of cases, preceded by chest pain (86.8%) and anaphylaxis (53%) (3, 39, 40). Indeed, the latter event may be particularly true especially if the blood pressure is low as in the described patient.

Skin prick tests could be useful in demonstrating IgE-mediated hypersensitivity (41). However, especially for some drugs, in case of negative results is not possible to completely rule out the diagnosis of hypersensitivity. Interestingly, skin tests themselves can induce KS and caution has to be paid to carry them out, especially when the diagnostic performance for the specific allergen is suboptimal (42). Unfortunately, serum tryptase dosage was not performed in our patient. However, although positive serum tryptase could be a useful biomarker in case of anaphylaxis and KS, negative results cannot rule them out completely (2).

In this case we highlighted the importance of recognition of KS in pediatric patients, differentiating this condition from other ones associated with acute ventricular systolic disfunction. Firstly, a hypersensitivity reaction should be taken into consideration as a potential cause of an acute coronary syndrome; secondly we pointed out the critical role of observing closely the patient, as the situation could evolve unpredictably to a cardiopulmonary emergency. Moreover, when a myocarditis or another cause are suspected, they benefit from a specific management (22). In the context of diagnostic investigations, in addition to ECG and echocardiography, cardiac MRI and coronary CT scan were performed, which were fundamental to assess our patient. In addition, his blood investigations did not show inflammatory markers increase.

In the present report, after the management of the acute phase, the stable clinical condition and the rapid improvement of systolic function allowed us to avoid the administration of specific drugs.

## Conclusion

In conclusion, we described one of the first pediatric cases of KS related to anesthetic medications. Cases reported of this syndrome are increasing progressively in the literature, and more and more drugs/agents have been identified as potential triggers of this clinical condition. The treatment of KS could be challenging, and it is mainly based on the treatment of the allergic reaction and of the cardiological clinical manifestations. In children, this syndrome has been only described in isolated case reports or small case series. Thus, it appears critical to report new cases of KS in children to increase the awareness of this disease in pediatric healthcare workers so as to enhance its early recognition and optimal therapeutic strategy. Furthermore, it appears of paramount importance the implementation of universal guidelines accepted by allergology and cardiology societies, in order to standardize the management of pediatric and adult patients with KS. Finally, a close collaboration between pediatric allergists and cardiologists seems fundamental for an optimal multidisciplinary patient care.

## Data Availability Statement

The original contributions presented in the study are included in the article/supplementary material, further inquiries can be directed to the corresponding author.

## Ethics Statement

Written informed consent was obtained from the minor(s)' legal guardian/next of kin for the publication of any potentially identifiable images or data included in this article.

## Author Contributions

GC, MG, LDS, and ST conceptualized the work. GC, MG, IK, FM, SF, LDS, and ST drafted the manuscript. GC, MG, FM, CR, GS, GBC, SF, EN, GI, LDS, and ST performed the investigations. GC, MG, IK, FM, CR, GS, GBC, SF, EN, GI, LDS, and ST critically revised the manuscript. All authors approved the final version of the manuscript as submitted and agreed to be accountable for all aspects of the work.

## Conflict of Interest

The authors declare that the research was conducted in the absence of any commercial or financial relationships that could be construed as a potential conflict of interest.

## Publisher's Note

All claims expressed in this article are solely those of the authors and do not necessarily represent those of their affiliated organizations, or those of the publisher, the editors and the reviewers. Any product that may be evaluated in this article, or claim that may be made by its manufacturer, is not guaranteed or endorsed by the publisher.
